# The secretome of *Verticillium dahliae* in collusion with plant defence responses modulates Verticillium wilt symptoms

**DOI:** 10.1111/brv.12863

**Published:** 2022-04-27

**Authors:** Dan‐Dan Zhang, Xiao‐Feng Dai, Steven J. Klosterman, Krishna V. Subbarao, Jie‐Yin Chen

**Affiliations:** ^1^ State Key Laboratory for Biology of Plant Diseases and Insect Pests, Institute of Plant Protection Chinese Academy of Agricultural Sciences Beijing 100193 China; ^2^ United States Department of Agriculture, Agricultural Research Service, Crop Improvement and Protection Research Unit Salinas CA 93905 USA; ^3^ Department of Plant Pathology University of California Davis CA 95616 USA

**Keywords:** vascular pathogen, *Verticillium dahliae*, secretome, toxins, vascular occlusion, Verticillium wilt.

## Abstract

*Verticillium dahliae* is a notorious soil‐borne pathogen that enters hosts through the roots and proliferates in the plant water‐conducting elements to cause Verticillium wilt. Historically, Verticillium wilt symptoms have been explained by vascular occlusion, due to the accumulation of mycelia and plant biomacromolecule aggregation, and also by phytotoxicity caused by pathogen‐secreted toxins. Beyond the direct cytotoxicity of some members of the secretome, this review systematically discusses the roles of the *V. dahliae* secretome in vascular occlusion, including the deposition of polysaccharides as an outcome of plant cell wall destruction, the accumulation of fungal mycelia, and modulation of plant defence responses. By modulating plant defences and hormone levels, the secretome manipulates the vascular environment to induce Verticillium wilt. Thus, the secretome of *V. dahliae* colludes with plant defence responses to modulate Verticillium wilt symptoms, and thereby bridges the historical concepts of both toxin production by the pathogen and vascular occlusion as the cause of wilting symptoms.

## I. INTRODUCTION

*Verticillium dahliae* is a well‐known hemi‐biotrophic soil‐borne pathogen that infects over 200 hosts and causes billions of dollars in losses annually (Inderbitzin & Subbarao, 2014). The pathogen attacks plants through the roots and colonizes the unique ecological niche of the plant xylem, resulting in Verticillium wilt, the symptoms of which include foliar wilting, chlorosis, stunting, necrosis and vein clearing (Fradin & Thomma, 2006; Chen *et al*., 2021). Over the past 60 years, two main mechanisms that may explain the induced wilt symptoms, i.e. vascular occlusion and through the involvement of toxins have been advanced in the literature (Fradin & Thomma, 2006; Báidez *et al*., 2007). Following successful infection of the plant, *V. dahliae* reaches the xylem and increases its biomass concurrent with plant defence responses that aim to restrict the pathogen, including the production of tyloses and accumulation of a colloidal matrix (Robb, Powell & Street, 1989; Báidez *et al*., 2007; Yadeta & Thomma, 2013). This eventually leads to vascular occlusion and plant wilting. An alternative view argues that the pathogen delivers toxins, which in this case refers broadly to a wide‐array of molecules and complex compounds such as large polysaccharides, protein‐lipopolysaccharides, glycoproteins, and enzymes including some that act as effectors that induce plant wilting (Fradin & Thomma, 2006; de Sain & Rep, 2015). Both hypotheses are equally credible (Pegg & Brady, 2002; Fradin & Thomma, 2006; Báidez *et al*., 2007; de Sain & Rep, 2015), and in fact, the two are tightly interlinked as the secretome, including these toxins, colludes with plant defence responses to cause Verticillium wilt symptoms. Here, we review recent findings and the mechanistic actions of the secretome of *V. dahliae*, which causes Verticillium wilt symptoms *via* both a contribution to vascular occlusion and *via* cytotoxicity.

### (1) The role of vascular occlusion in Verticillium wilt

Following successful breaching of the endodermis, *V. dahliae* generally is confined to the fluid environment of the xylem cells and exerts its effects on host physiology indirectly (Yadeta & Thomma, 2013). Unlike prokaryotic vascular pathogens that break out of the vascular system and spread indiscriminately in the root and shoot parenchyma, *V. dahliae* seldom leaves the confines of the xylem until the death of the surrounding tissue or the host (Pegg & Brady, 2002). Key steps in the pathogenesis of *V. dahliae* include germination of microsclerotia, hyphal penetration of the root epidermis, and growth in the cortex that crosses the endodermis finally breaching the highly structured and rigid secondary xylem walls to enter vessels of the root (Zhao *et al*., 2014). Once in the xylem, the pathogen produces conidia, which germinate, ramify and penetrate adjacent vascular cells to continue colonization (Fradin & Thomma, 2006; Yadeta & Thomma, 2013).

Since *V. dahliae* enters the xylem tissue, it can be trapped in the pit cavities at the xylem perforation plates. Because the nutritional value of the xylem fluid is limited, the pathogen draws its nutrition by degrading the vascular cell wall using an abundance of diverse extracellular enzymes encoded within its genome (Klosterman *et al*., 2011; Chen *et al*., 2016). The insufficient degradation of the xylem walls and pit membranes causes the deposition of polysaccharides (Fradin & Thomma, 2006; Kubicek, Starr & Glass, 2014). In addition, this triggers plant defences to produce tyloses and other biomacromolecules (resins, gums, gels, etc.) from neighbouring parenchyma cells, which act as a defensive barricade to restrict pathogen spread (Benhamou, 1995; Yadeta & Thomma, 2013). Analyses of the plant vascular system using electron microscopy during Verticillium wilt of tomato (*Lycopersicon esculentum* L.) has indicated that terminal occlusion leading to sealing of entire veins is a limiting factor preceding the irreversible appearance of vascular wilt (Robb, Powell & Street, 1989). Thus, the pathogen rapidly proliferates, accompanied by continuous deposition of polysaccharides and plant defence‐driven structures and biomacromolecules (tyloses, suberin, etc.) that interfere with the flow of water and nutrition, ultimately leading to plant wilting (Pegg & Brady, 2002; Báidez *et al*., 2007; Yadeta & Thomma, 2013).

### (2) The role of toxins in Verticillium wilt

The concept that toxins could underlie *Verticillium‐*induced foliar wilting was first proposed from results obtained using components from culture filtrates that induced wilt symptoms (Stoddart & Carr, 1966; Meyer & Dubery, 1993). Since then, efforts have been made to elucidate the role of toxins in the mechanisms of foliar wilting (Pegg & Brady, 2002). While some evidence linking toxins to foliar wilting is undeniable, other investigations have yielded ambiguous results (Fradin & Thomma, 2006). For instance, a low molecular weight phytotoxic polypeptide that exhibited toxicity to potato (*Solanum tuberosum* L.) leaves was detected using immunofluorescent antibodies in the xylem of infected potato stems (Nachmias, Buchner & Burstein, 1985). A similar toxin induced the typical foliar wilting when injected into *Arabidopsis thaliana* seedlings (Jing, Fan & Wu, 2005). Incremental progress in understanding the toxins involved has occurred with advances in genomics and metabolomics technologies, and some toxin components have been verified, including a range of proteins with enzymatic or effector activity (Klimes *et al*., 2015), metabolites (Zhang *et al*., 2016; Zhang *et al*., 2019a), and volatile compounds (Li & Kang, 2018). Proteins secreted from *V. dahliae* that serve as toxins have been shown to play critical roles in its pathogenesis resulting in foliar wilting through plant cell wall destruction, manipulation of host responses, or cytotoxicity (de Sain & Rep, 2015; Klimes *et al*., 2015; Chen *et al*., 2016). The secreted proteins have been suggested to target various sites, including the cell wall, plasma membrane, and other intracellular components (Meyer & Dubery, 1993), and also to induce changes in the microfilaments and microtubules in plant cells (Zhao *et al*., 2020), leading to rapid cell lesions.

## II. FUNCTIONS OF THE *VERTICILLIUM DAHLIAE* SECRETOME

**Fig. 1:**
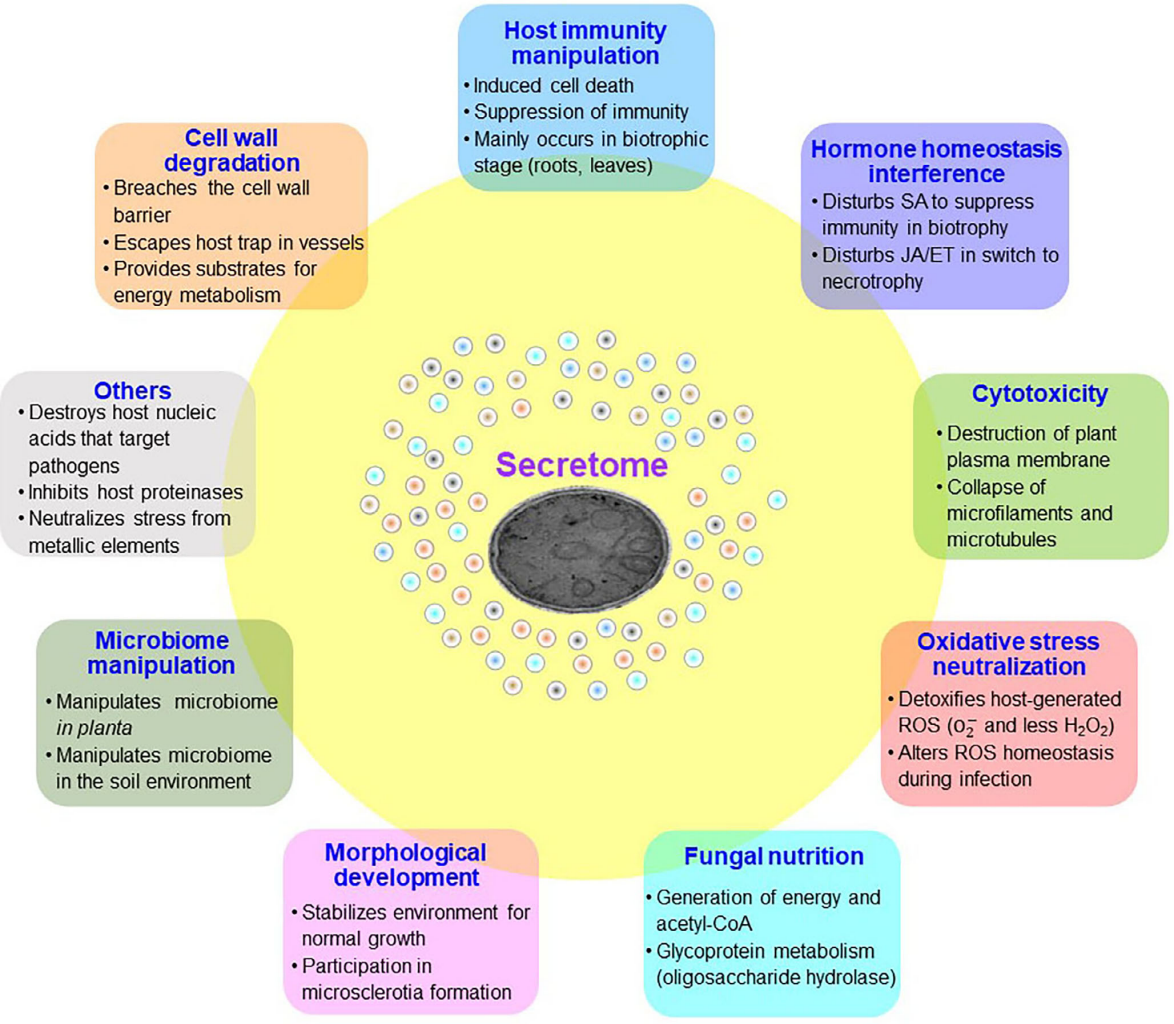
Versatile biological functions of the secretome in *Verticillium dahliae*. Acetyl‐coA, acetyl co‐enzyme A; ET, ethylene; JA, jasmonic acid; ROS, reactive oxygen species; SA, salicylic acid.

Fungal pathogens secrete numerous proteins (i.e. their secretome) to modulate plant defences by altering host cellular structure and physiology, and to facilitate colonization (Hogenhout *et al*., 2009). Since *V. dahliae* mainly resides in the vascular tissue during its disease cycle, understanding the role of the secretome in contributing to vascular occlusion or producing toxins could offer a better understanding of the mechanisms that underlie foliar wilting. Genomics‐driven research has shown that *V. dahliae* encodes more than 700 potential secreted proteins (Klosterman *et al*., 2011; Chen *et al*., 2018), and hundreds are delivered into the extracellular space with generalist functions (Chen *et al*., 2016). Currently, many secreted proteins have been shown to contribute to pathogenesis, resulting in Verticillium wilt *via* diverse modes of action (Table 1). Thus, *V. dahliae* employs its secretome to degrade plant cell walls, manipulate host immunity, exert cytotoxicity, neutralize host oxidative stress, compete with its host for nutrients, and even to affect the host microbiome to facilitate Verticillium wilt development (Fig. 1).

**Table 1:** Description of some secreted proteins involved in various biological processes of *Verticillium dahliae*.

Secreted protein	Function description	Cell wall degradation	Host immunity manipulation	Hormone homeostasis Interference	Cytotoxicity	Oxidative stress neutralization	Fungal nutrition	Morphological development	Microbiome manipulation	Others	Host molecular target	Reference
VD18.5	Phytotoxic protein				**√**							Palmer, Saleeba & Lyon (2005)
VDH1	Class II hydrophobin gene							**√**				Klimes & Dobinson (2006)
VdEg‐1	Endoglucanase 1	**√**						**√**				Maruthachalam *et al*. (2011)
VdAve1	Avirulence gene of race 1		**√**	**√**					**√**		Ve1	de Jonge *et al*. (2012); Snelders *et al*. (2020)
VdNLP1	Necrosis‐ and ethylene‐inducing‐like protein				**√**			**√**			GIPC sphingolipids	Zhou *et al*. (2012); Santhanam *et al*. (2013)
VdNLP2	Necrosis‐ and ethylene‐inducing‐like protein				**√**						GIPC sphingolipids	Zhou *et al*. (2012); Santhanam *et al*. (2013)
VdSSP1	Cell wall degradation related protein	**√**					**√**					Liu *et al*. (2013)
VdISC1	Isochorismatase		**√**	**√**							Isochorismate	Liu *et al*. (2014)
VdPL3.1	Pectin lyase	**√**										Chen *et al*. (2016)
VdPL3.3	Pectin lyase	**√**										Chen *et al*. (2016)
Vd2LysM	Chitin‐binding lysin motif		**√**					**√**			Plant chitinases	Kombrink *et al*. (2017)
VdASP F2	Allergen Asp F2‐like protein							**√**				Xie, Li & Yang (2017)
VdCBM1	Cellulose binding module 1	**√**	**√**	**√**								Gui *et al*. (2017, 2018); Wang *et al*. (2020a)
VdCP1	Cerato‐platanin protein 1		**√**	**√**							Plant chitinases	Zhang *et al*. (2017a)
VdEG1	GH12 domain‐containing protein	**√**	**√**	**√**								Gui *et al*. (2017)
VdEG3	GH12 domain‐containing protein	**√**	**√**	**√**								Gui *et al*. (2017)
VdSCP7	Small cysteine‐rich protein		**√**	**√**								Zhang *et al*. (2017b)
VdPEL1	Pectate lyase	**√**	**√**	**√**								Yang *et al*. (2018)
VdPL1	Polysaccharide lyase	**√**										Zhang *et al*. (2018)
VdCUT11	Cutinase	**√**	**√**	**√**								Gui *et al*. (2018)
PevD1	Alt a 1 family protein		**√**	**√**							GhPR5, ORE1	Zhang *et al*. (2021); Liang *et al*. (2018)
VdSCP41	Small cysteine‐rich protein		**√**	**√**							CBP60g and SARD1	Qin *et al*. (2018)
VdOCH1	Alpha‐1, 6‐mannosyltransferase						**√**	**√**				Zhang *et al*. (2019b)
VdPDA1	Polysaccharide deacetylase		**√**	**√**							Chitin oligomer	Gao *et al*. (2019)
VdSSEP1	Secretory Ser protease 1		**√**	**√**						**√**	Plant chitinases	Han *et al*. (2019)
VdSCP27	Small cysteine‐rich protein		**√**	**√**								Wang *et al*. (2020a)
VdSCP113	Small cysteine‐rich protein		**√**	**√**								Wang *et al*. (2020a)
VdSCP126	Small cysteine‐rich protein		**√**	**√**								Wang *et al*. (2020a)
VdSOD3	Superoxide dismutase					**√**						Tian *et al*. (2020)
VdAMP2	Antimicrobial effector								**√**			Snelders *et al*. (2020)
VdSOD5	Superoxide dismutase					**√**						Tian *et al*. (2021a)
VdSOD1	Cu/Zn superoxide dismutase					**√**						Tian *et al*. (2021b)
Av2	Avirulence gene of race 2		**√**								V2	Chavarro‐Carrero *et al*. (2021)
VdEIX3	Ethylene‐inducing xylanase	**√**	**√**	**√**							EIX2	Yin *et al*. (2021)
VdXyn4	Xylanase	**√**	**√**	**√**	**√**		**√**					Wang *et al*. (2021a)
VDAL	Asp f2‐like protein		**√**								PUB25/26	Ma *et al*. (2021)
VdAMP3	Antimicrobial effector								**√**			Snelders *et al*. (2021)
VdR3e	Avirulence gene of race 3		**√**									Wang *et al*. (2021b)
VdRTX1	Ribonuclease		**√**							**√**		Yin *et al*. (2022)

CBP60g, calmodulin binding protein 60 family member g; EIX2, leucine‐rich repeat receptor‐like protein; GIPC, glycosylinositol phosphorylceramide; ORE1, A senescence‐associated NAC transcription factor; PR5, pathogenesis‐related protein 5 (PR5)‐like protein; PUB25/26, plant U‐box 25/26; SARD1, systemic acquired resistance deficient 1; Ve1, leucine‐rich repeat receptor‐like protein.

**Fig. 2:**
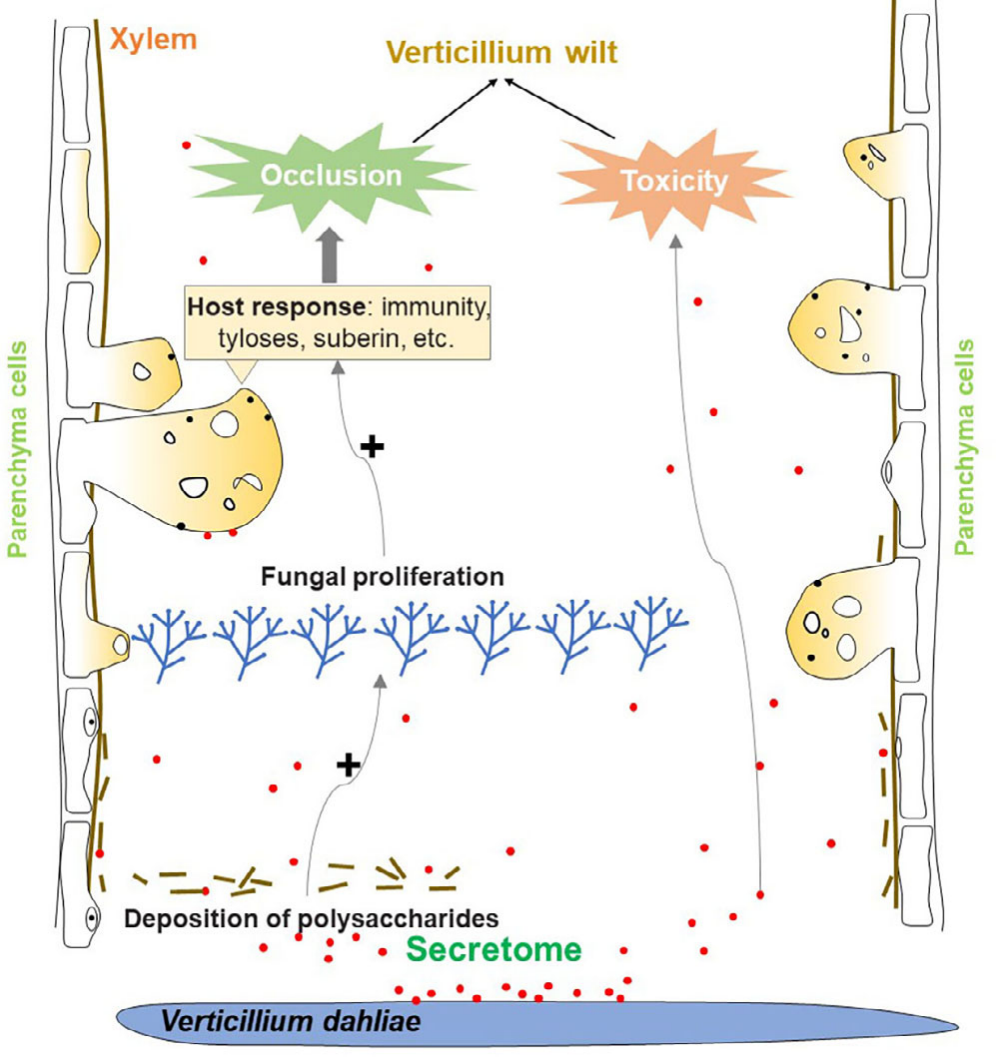
The secretome of *Verticillium dahliae* causes vascular occlusion which results in Verticillium wilt. Its secretome contributes to vascular occlusion by promoting fungal proliferation and polysaccharide deposition, and by inducing host responses including tyloses formation and suberin production. The secretome also contributes to Verticillium wilt through cytotoxicity.

*Verticillium dahliae* employs its secretome to proliferate in the low‐nutrient confines of the xylem. Its secretome encodes a large arsenal of enzymes that allow the complete degradation of many plant polysaccharides (Klosterman *et al*., 2011). These processes inevitably induce xylem collapse and the deposition of polysaccharides. The host plants respond by producing biomacromolecules (gums, gels, and other resins) and tyloses as defensive structures which protrude into the vascular tissue to block progress of the pathogen. Together, the massive propagation of hyphae, the deposition of polysaccharides and the production of biomacromolecules by the plant cause xylem occlusion and plant wilting (Fig. 2).

### (1) Proliferation in the plant xylem

#### (a) Cell wall degradation

During the infection process, *V. dahliae* must break through plant root cell wall barriers to reach the xylem. During this process, *V. dahliae* produces an array of cell wall degrading enzymes (CWDEs) (Kubicek, Starr & Glass, 2014). Some of these CWDEs destroy the highly structured and rigid secondary xylem walls, and also enable breaches of the pectin‐enriched pit cavities or the vascular end walls (Zhao *et al*., 2014). Additional secreted enzymes include two members of the glycoside hydrolase 12 family, the endoglucanases VdEG1 and VdEG3, which participate in cellulose/hemicellulose degradation (Gui *et al*., 2017) similar to the CWDEs involved in degradation of the plant cuticle and xylan (Gui *et al*., 2018; Yin *et al*., 2021; Wang *et al*., 2021a). Furthermore, transcription regulators of CWDEs such as the sucrose non‐fermenting protein kinase VdSNF1 (Tzima *et al*., 2021) and fungal‐specific transcription factor VdFTF1 (Zhang *et al*., 2018) regulate CWDEs expression or secretion, and significantly contribute to pathogenesis. The superabundance of secreted carbohydrate‐active enzymes suggests an extraordinary capacity of *V. dahliae* to degrade plant cell wall barriers during early infection stages and to degrade plant xylem walls during proliferation in these vessels.

#### (b) Scavenging host reactive oxygen species

Plants respond to pathogen attack with a transient burst of reactive oxygen species (ROS) that play a central role in plant immune responses (Qi *et al*., 2017). Pathogens can counteract this by secreting ROS‐scavenging proteins to neutralize or degrade ROS, such as superoxide dismutases (SODs), catalases, and peroxidases (Broxton & Culotta, 2016; Khademian & Imlay, 2021). Comparative analyses of the *V. dahliae* secretome have revealed that many secreted proteins with ROS‐scavenging and oxidative stress response functions are activated when the pathogen is cultured on host tissue or under nutrient‐starvation conditions (El‐Bebany, Rampitsch & Daayf, 2010; Chu *et al*., 2015; Chen *et al*., 2016). *V. dahliae* secretes SODs to detoxify host‐generated extracellular ROS, contributing significantly to virulence during plant infection (Tian *et al*., 2020, 2021a,b). *V. dahliae* ROS production facilitates penetration peg formation during the initial colonization of cotton (*Gossypium hirsutum* L.) roots (Zhao, Zhou & Guo, 2016). Thus, the deployment of ROS during infection combined with counter‐provisions for neutralization of host‐generated oxidative stress is key to successful infection and proliferation in the vascular tissue of host plants.

#### (c) Suppressing host immunity

**Fig. 3:**
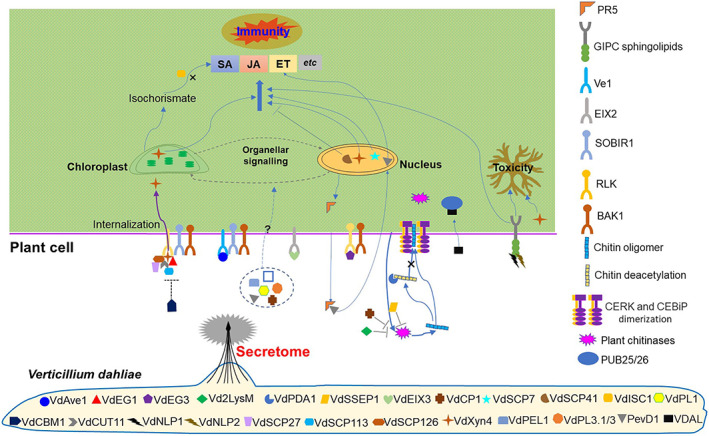
*Verticillium dahliae* employs its secretome to manipulate host immunity and interfere with hormone homeostasis. See Table 1 for *V. dahliae* protein names. VdAve1 is recognized as an avirulence determinant by tomato plants that carry the corresponding Ve1 immune receptor. VdEG1 and VdEG3 are glycoside hydrolase 12 proteins that trigger immunity dependent on the LRR‐RLPs/SOBIR1/BAK1 and LRR‐RLKs/BAK1 complexes, respectively. Vd2LysM binds long‐ or short‐chain chitin oligomers and prevents degradation of chitin by plant chitinase. VdPDA1 directs deacetylation of chitin oligomers and inhibits perception by host LysM‐containing receptors, thus avoiding ligand‐triggered immunity. VdSSEP1 hydrolyses cotton Chi28 directly, inhibiting the production of chitin oligomers. VdEIX3 exhibits immunity‐inducing activity in *Nicotiana benthamiana*, recognized by the leucine‐rich repeat receptor‐like protein NbEIX2. VdCP1 protects the *V. dahliae* cell wall from chitinase degradation. VdSCP7 targets the host nucleus to modulate plant immunity. VdSCP41 targets the plant‐specific transcription factors CBP60g and SARD1 to modulate immunity. VdISC1 disrupts the plant salicylate metabolism pathway by suppressing the transformation from isochorismate to salicylic acid. VdPL1 plays a virulence function during infection of cotton. VdCBM1 suppresses VdEG1‐, VdEG3‐, VdSCP27‐, VdSCP113‐, VdSCP126‐, VdCUT11‐ and VdXyn4‐induced cell death and some PAMPs‐triggered immunity in *N. benthamiana*. VdCUT11 induces plant defence responses in *N. benthamania* in a BAK1‐ and SOBIR1‐dependent manner. VdNLP1 and VdNLP2 are GIPC sphingolipids that act as NLP toxin receptors; NLPs form complexes with terminal monomeric hexose moieties of GIPCs and insert into the plant plasma membrane, causing cell lysis. VdSCP27, VdSCP113 and VdSCP126 induce defence responses in *N. benthamania* in a BAK1‐ and SOBIR1‐dependent manner. VdXyn4 plays a cytotoxic function and induces a necrosis phenotype in *N. benthamania*, depending on simultaneous localization to the nuclei and chloroplasts in a BAK1‐ and SOBIR1‐dependent manner. VdPEL1 exhibits pectin hydrolytic activity and induces cell death in plants. VdPL3.1/3 have virulence functions during infection of cotton. PevD1 induces ethylene biosynthesis by directly binding to ORE1. VDAL protects transcription factor MYB6 from degradation by interacting with the E3 ligases PUB25 and PUB26 to enhance Verticillium wilt resistance. BAK1, LRR‐RLK BRI1‐associated kinase‐1; CBP60g, calmodulin binding protein 60 family member g; CEBiP, chitin‐elicitor binding protein; CERK, receptor chitin elicitor receptor kinase; Chi28, chitinase 28; EIX2, leucine‐rich repeat receptor‐like protein; ET, ethylene; GIPC, glycosylinositol phosphorylceramide; JA, jasmonic acid; LRR, leucine‐rich repeat; LysM, lysin motif; MYB6, MYB domain protein 6; NLP, necrosis‐ and ethylene‐inducing‐like protein; ORE1, A senescence‐associated NAC transcription factor; PAMP, pathogen‐associated molecular pattern; PR5, pathogenesis‐related protein 5 (PR5)‐like protein; PUB25/26, plant U‐box 25/26; RLK, receptor‐like kinase; RLP, receptor‐like protein; SA, salicylic acid; SARD1, systemic acquired resistance deficient 1; SOBIR1, LRR‐RLK suppressor of BIR1‐1; Ve1, leucine‐rich repeat receptor‐like protein.

The ability to secrete effector proteins that can enter plant cells and manipulate host immunity is a key determinant of a successful plant pathogen (He *et al*., 2020). In *V. dahliae*, the role of effectors in manipulating host immunity has been studied in detail (Fig. 3). To enable successful infection and proliferation in host plants, *V. dahliae* has evolved a range of effector proteins that inhibit host immune responses (Ding & Redkar, 2018), including inactivation of the salicylic acid (SA) pathway by isochorismate synthase VdIsc1 (Liu *et al*., 2014), cellulose‐binding module family 1‐member (VdCBM1) (Gui *et al*., 2017, 2018; Wang *et al*., 2021b), small cysteine‐rich protein VdSCP41 (Qin *et al*., 2018), lysin motif (LysM) protein Vd2LysM (Kombrink *et al*., 2017), secretory serine protease 1 (VdSSEP1) (Han *et al*., 2019) and polysaccharide deacetylase (VdPDA1) (Gao *et al*., 2019) (Fig. 3; Table 1). For instance, VdPDA1 deacetylates chitin oligomers; since the *N*‐acetyl group contributes to host LysM‐containing receptor recognition for ligand‐triggered immunity, the action of VdPDA1 renders the host susceptible to *V. dahliae* by evading host surveillance through inactivation of a chitin‐triggered host immune response (Gao *et al*., 2019). Together, these effectors mainly function in the early stages of infection and employ a variety of strategies to overcome immunity to facilitate pathogen colonization in roots.

#### (d) Fungal nutrition

Fungi secrete many plant CWDEs such as cellulase, hemicellulase, and pectin‐degrading enzymes that act on plant tissues to produce monomeric and/or small polymeric sugars that are subsequently transported into the fungal cell *via* membrane‐bound transporters (Glass *et al*., 2013). In this way, *V. dahliae* obtains nutrition for proliferation in the vascular tissue. Many secreted carbohydrate‐active enzymes from *V. dahliae* catalyse the release of nutrients as the degradation products of cell walls (Klosterman *et al*., 2011; Xiong, Wang & Tian, 2015; Chen *et al*., 2016) (Table 1, Fig. 3), some of which function in the generation of energy and acetyl‐CoA during nutrient starvation (Chu *et al*., 2015; Xiong, Wang & Tian, 2015). The *V. dahliae* alpha‐1,6‐mannosyltransferase (VdOCH1) plays a crucial role in *N*‐linked oligosaccharide glycoprotein metabolism (Zhang *et al*., 2019b). The abundant pectate lyases secreted by *V. dahliae* are thought to have key roles in its adaptation to its vascular niche (Klosterman *et al*., 2011).

#### (e) Microbiome manipulation

Although pathogen effectors are typically considered to act exclusively through direct host immunity manipulation, plant pathogens also utilize effectors to target the host microbiota in order to facilitate niche colonization (Rovenich, Boshoven & Thomma, 2014; Snelders *et al*., 2018). Recent work (Snelders *et al*., 2020, 2021) suggests that *V. dahliae* exploits secreted proteins to manipulate the host microbiome through its antibacterial or antifungal properties, to promote infection. The *V. dahliae* avirulence effector (VdAve1) displays antimicrobial activity and facilitates colonization of tomato and cotton through the selective manipulation of the host microbiota in the roots, as well as in the xylem by suppressing antagonistic bacteria. The antimicrobial protein VdAMP2, is exclusively expressed by *V. dahliae* when in the soil and exerts antibacterial activity that contributes to niche establishment (Snelders *et al*., 2020). Thus, VdAMP2 and VdAve1 likely have complementary functions for optimal soil and host colonization. Another ancient antimicrobial protein (VdAMP3) is specifically expressed to ward off fungal niche competitors during resting structure formation in senescing host mesophyll tissues (Snelders *et al*., 2021), allowing *V. dahliae* to tailor the expression of microbiome‐manipulating effectors based on the microbiota that it encounters during different stages of the disease cycle. This promotes *V. dahliae* colonization and proliferation in host plants to accelerate symptom development.

### (2) Xylem wall collapse and deposition of polysaccharides

Compared to many fungi, the genome of *V. dahliae* encodes a significant expansion of gene families involved in plant cell wall degradation, and specific members of this repertoire may be essential for adaptation to its vascular niche (Klosterman *et al*., 2011). For example, among plant cell wall polysaccharides, pectin has the highest structural and functional complexity (Mohnen, 2008; Harholt, 2010), with four major structural classes: homogalacturonan (HG), rhamnogalacturonan I (RG‐I), xylogalacturonan (XG), and rhamnogalacturonan II (RG‐II). Enzymes required for efficient degradation of pectin (Martens‐Uzunova & Schaap, 2009) include polygalacturonases and rhamnogalacturonases (glycoside hydrolases family 28, GH28), polysaccharide lyases (pectin and pectate lyases, PL1, PL2, and PL3), and rhamnogalacturonan lyases (PL4), as well as pectin methylesterases (carbohydrate esterase family 8, CE8), pectin acetylesterases (CE12 and CE13), and rhamnogalacturonan acetylesterases (CE12). Among all sequenced fungal genomes, *V. dahliae* has one of the most complete repertoires of polysaccharide lyases and associated glycoside hydrolases for the breakdown of complex pectin (Klosterman *et al*., 2011). Experimental evidence indicates that the *V. dahliae* polysaccharide lyase VdPL1 (Zhang *et al*., 2018), pectate lyase VdPEL1 (Yang *et al*., 2018), pectin lyases VdPL3.1 and VdPL3.3 (Chen *et al*., 2016), and the pathotype‐specific secretory protein VdSSP1 (Liu *et al*., 2013) are all involved in virulence and pectin degradation. Since pectin is enmeshed with other polysaccharides to form plant cell walls, the result is that incompletely degraded and intermediate polysaccharides contribute directly to vascular occlusion (Kubicek, Starr & Glass, 2014). In addition, in response to infections, pectin gels are usually released into the xylem (Rioux *et al*., 1998; Clérivet *et al*., 2000). Similarly, starch hydrolysis also contributes to vessel occlusion related to wilt symptoms in olive (*Olea europaea* L.) stems of susceptible cultivars infected by *V. dahliae* (Trapero *et al*., 2018). Thus, while degradation of the xylem walls and pit membranes by *V. dahliae* CWDEs supplies nutrients for pathogen proliferation in the low‐nutrient confines of the xylem, it also causes xylem collapse and blockage by deposition of insufficiently degraded polysaccharides.

### (3) Induction of host response that aggravate vascular occlusion

While secretion of an array of CWDEs is required for breaching cell wall barriers and nutrient acquisition, *V. dahliae* also must overcome host defence responses. It achieves this with a combination of secreted effectors and interference with host hormone signalling, both of which contribute to vascular occlusion and wilt symptoms. Among plant hormones, SA, jasmonic acid (JA) and ethylene (ET) are all significant in the hormonal regulation of plant immune responses (Shigenaga & Argueso, 2016). SA has been implicated in the protection of plant tissues during the initial biotrophic phase of *V. dahliae* infection, while JA is more prominent following the establishment of *V. dahliae* in the xylem and its switch to a necrotrophic lifestyle (Dhar *et al*., 2020). ET activates defence responses to limit spread of the pathogen during the early stages of *V. dahliae* infection; but during subsequent colonization and the switch to the necrotrophic phase, ET can facilitate establishment of the pathogen and accelerate the progression of Verticillium wilt (Dhar *et al*., 2020). The *V. dahliae* secretome interferes with the intricate and delicate balance of phytohormones during *V. dahliae*–plant interactions, regulating the immune responses in a synergistic or antagonistic manner that contributes to Verticillium wilt progression (Dhar *et al*., 2020). Manipulation of SA–JA signalling has been demonstrated for several known effectors in *V dahliae*, including four VdSCPs (Wang *et al*., 2012; Wang *et al*., 2020a), the cutinase VdCUT11 (Gui *et al*., 2018), the necrosis‐ and ethylene‐inducing‐like protein VdNLP (Wang *et al*., 2004), the endoglucanases VdEG1 and VdEG3 (Gui *et al*., 2017), VdSCP7 (Zhang *et al*., 2017a,b) and the xylanase VdXyn4 (Wang *et al*., 2021a,b) (Fig. 3). Additionally, VdNLP involved in ET signalling aids in the establishment of the pathogen and accelerates the progression of Verticillium wilt and death of the host (Wang *et al*., 2004; Dhar *et al*., 2020), and PevD1 induces ET biosynthesis by directly binding to the senescence‐associated NAC transcription factor ORE1 (Zhang *et al*., 2021).

In response to *V. dahliae* infection and colonization, the plant may mount a rapid defence including deposition of coating materials such as suberin on vascular cell walls, forming a barrier to fungal penetration and horizontal spread (Robb, Powell & Street, 1989). At this stage, infection can result in vessel occlusion by gums, gels, tyloses and other deposited resins secreted by the neighbouring parenchyma cells (Robb *et al*., 1979; Benhamou, 1995). In addition, plant hormone levels can be manipulated by effectors secreted by pathogens, influencing plant responses to the pathogen, including vascular occlusion. During plant–pathogen interactions, many phytohormones regulate the formation of coating materials on vascular cell walls, including ET, which is involved in lignin and suberin biosynthesis, and tyloses formation, while abscisic acid coordinates suberin deposition, JA contributes to lignin biosynthesis and tyloses formation, and SA can abolish the induction of tyloses by JA (Sun *et al*., 2007; Leśniewska *et al*., 2017; Hu *et al*., 2018; Liu *et al*., 2020; Wang *et al*., 2020b). Some of these regulatory mechanisms have been inferred in *V. dahliae*–plant interactions (Hu *et al*., 2019; Dhar *et al*., 2020). In *V. longisporum* infections, *Arabidopsis thaliana* plants can generate xylem *de novo* by transdifferentiation of the chloroplast‐containing bundle sheath cells into functional xylem elements, enhancing water transport, storage capacity, and drought tolerance (Reusche *et al*., 2012). Whether a similar mechanism exists during *V. dahliae*–plant interactions with a role in relieving plant wilt symptoms requires further investigation. Together, the secreted effectors from *V. dahliae* cause host immune responses and disturb the phytohormone balance contributing to vascular occlusion and the induction of Verticillium wilt symptoms (Fig. 2).

### (4) Toxicity

A cytotoxic function for all identified secreted proteins is difficult to demonstrate explicitly because a similar plant necrosis phenotype accompanies immunity‐ and cytotoxin‐induced necrosis. While some studies have shown that crude *Verticillium* culture extracts cause microfilaments and microtubules to collapse in plant cells (Zhao *et al*., 2020), it remains unclear whether it is toxic activity that directly causes necrosis. A prominent example of a fungal protein with cytotoxic activity is NLP purified from the culture filtrates of *Fusarium oxysporum* (Bailey, James & James, 1997). A NLP member identified from the mycelia of a *V. dahliae* strain pathogenic to cotton displayed the ability to cause foliar necrosis in various plant species (Wang *et al*., 2004). With the advent of genomics, seven additional members of the NLP family have been identified from the *V. dahliae* genome, two of which cause cellular necrosis (Zhou *et al*., 2012; Santhanam *et al*., 2013). The proposed mechanism of NLP‐mediated cell lysis is that glycosylinositol phosphorylceramide (GIPC) sphingolipids act as NLP receptors to form complexes with terminal monomeric hexose moieties of GIPCs that insert into the plant plasma membrane (Ottmann *et al*., 2009; Lenarčič *et al*., 2017) (Fig. 3). Additionally, a member of the xylanase family, VdXyn4 induces vein necrosis and plant wilting during advanced stages of infection, but not immunity‐triggered cell death, suggesting that it is a candidate toxicity factor (Wang *et al*., 2021a) (Fig. 3). Thus, constituents of the *V. dahliae* secretome may induce foliar wilting directly *via* toxicity, even if the definition of toxicity is narrow in this context.

## III. HOW THE SECRETOME COMPROMISES THE HOST TO CAUSE VASCULAR OCCLUSION AND VERTICILLIUM WILT

**Fig. 4:**
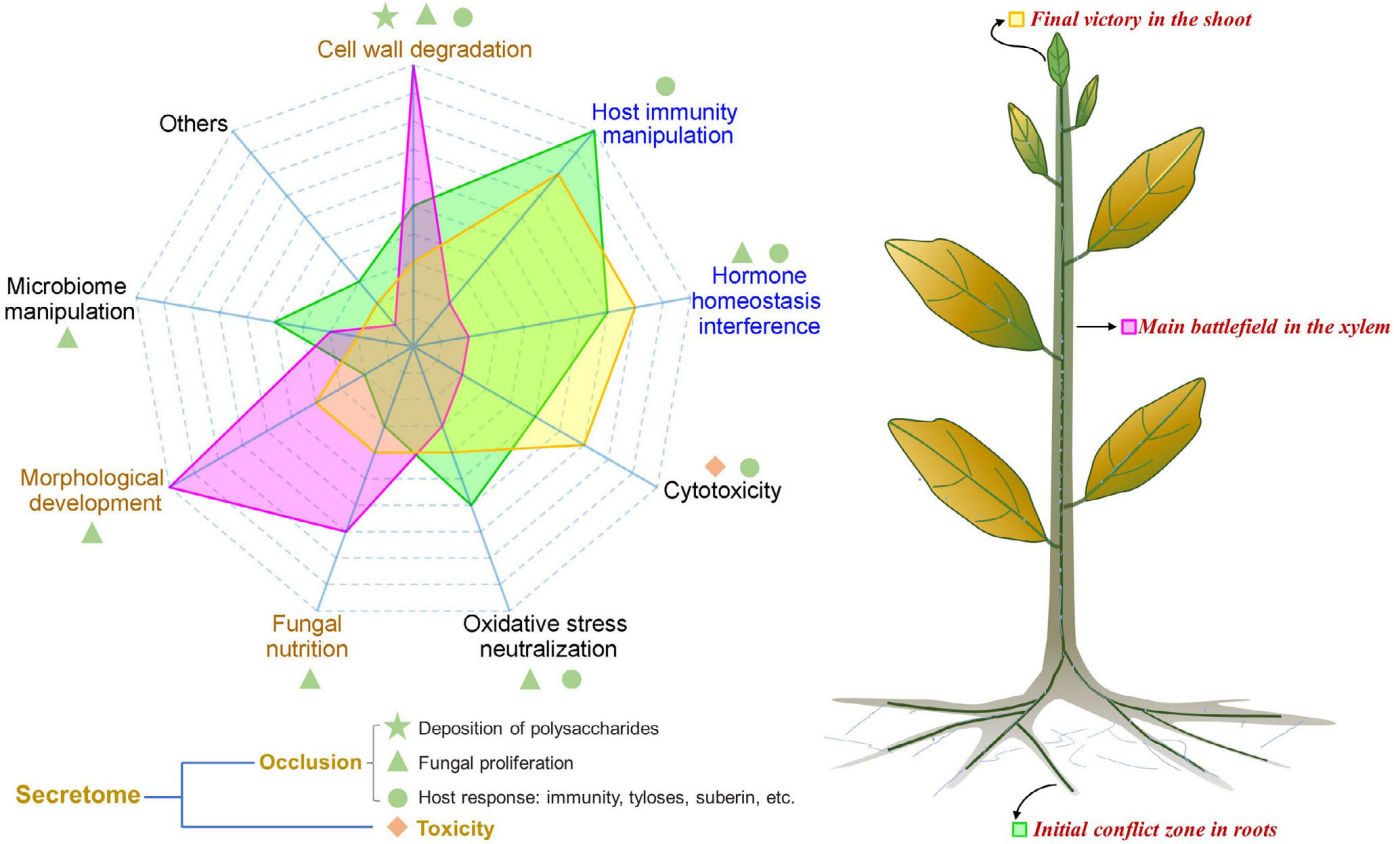
Intensity model of biological functions of the secretome at different stages of Verticillium wilt infection. Left: the main contributions of different functions of the secretome in polysaccharide deposition, fungal proliferation, host response and toxicity. Cell wall degradation, morphological development and fungal nutrition predation (shown in brown font) represent the main effects of the secretome in vessels; and host immunity manipulation and hormone interference (shown in blue font) represent the main effects of the secretome in roots and leaves. The green region shows the biological functions of the secretome that operate during the initial stages of the colonization, the pink region shows those operating when the fungus is present in the xylem, and the yellow region is for the final stage of infection when the pathogen has reached the leaves (see plant image on right).

The pathogenic status of *V. dahliae* is dynamic during the different stages of host infection (Fradin & Thomma, 2006), and the secretome plays a critical role in Verticillium wilt disease progression by both supplying toxins and contributing to vascular occlusion to cause irreversible and lethal plant wilting (Fig. 4).

### (1) Initial conflict zone in roots

*Verticillium dahliae* initially attacks plants through the root tip or through lateral roots by developing an infectious structure known as hyphopodium (Zhao, Zhou & Guo, 2016), which assists in establishing biotrophy (Fradin & Thomma, 2006). The hyphopodium develops a septin ring while piercing the root epidermis and cortical cells, that acts as a functional fungus–host penetration interface for the delivery of secreted fungal proteins (Zhou, Zhao & Guo, 2017). Subsequently, the secretome overcomes host defence responses by manipulating host immunity, interfering in hormone homeostasis, neutralization of oxidative stress, and cytotoxicity. The secreted CWDEs breach the plant cell walls to allow further penetration and proliferation (Fradin & Thomma, 2006). Even with these specialized tools, only a few invading hyphae successfully traverse into the vessel cells (Zhao *et al*., 2014). In addition, several CWDEs are involved in manipulating host immunity and altering hormone homeostasis (Table 1). Prior to penetration of plant roots, the rhizosphere environment is also crucial for *V. dahliae* survival and its eventual invasion. Here, effector proteins can suppress the growth of antagonistic bacteria (Snelders *et al*., 2020, 2021). Thus, ‘toxic’ activities of the *V. dahliae* secretome are critical for successful penetration, infection, and xylem colonization (Fig. 4).

### (2) The vascular battlefield

Once *V. dahliae* crosses the endodermis and reaches the xylem (generally 2–4 days), the hyphae begin to branch, develop terminal conidia and proliferate in the xylem (Fradin & Thomma, 2006). At this stage, the pathogen channels energy into vegetative growth resulting in hyphal extension and conidia formation in the xylem (Zhao *et al*., 2014). During this stage, *V. dahliae* uses its extensive range of CWDEs to survive in the barren environment of the xylem (Klosterman *et al*., 2011), and also secretes effectors with toxicity functions, for example VdXyn4 acts as a toxin during the colonization of vessels by destroying the host vascular system (Wang *et al*., 2021a). Concurrently, the host plants respond to the extensive sporulation and proliferation in the xylem by initiating rapid defence responses (Benhamou, 1995; Heinz *et al*., 1998; Pegg & Brady, 2002). In resistant hosts, colonization and proliferation of the pathogen are hindered by its compartmentalization in the vascular tissue. In susceptible hosts, the pathogen escapes these host traps by counteracting host defences *via* secreted effectors and hyphal growth that progresses through the occluded xylem (Fradin & Thomma, 2006). In this process, *V. dahliae* effectors also disturb the xylem microbiome (Snelders *et al*., 2020, 2021), further accelerating fungal proliferation and initiating a resistance response, that in turn leads to further xylem occlusion. Therefore, in the battle over control of the plant xylem, *V. dahliae* deploys its secretome to aid mycelial proliferation, resulting in release of incompletely degraded polysaccharides from the plant cell walls and induction of biomacromolecules from a host response, all of which contribute to vascular occlusion and the appearance of wilt symptoms (Fig. 4).

### (3) Final victory in the shoot

The successful proliferation of *V. dahliae* in the xylem results in two damaging effects: vascular occlusion blocking water and nutrient transport; and rapid pathogen transmission to the leaves *via* the vascular plumbing (Pegg & Brady, 2002; Fradin & Thomma, 2006). The pathogen can easily degrade the cell walls of leaf tissues since these are mainly coated with aliphatic biopolymers composed of cutin rather than suberin (Schreiber, 2010). To infect foliar cells, secreted proteins are released that manipulate host immunity and break hormone homeostasis (Liu *et al*., 2014; Gui *et al*., 2017; Gao *et al*., 2019). For example, *V. dahliae* induces leaf senescence and wilting by secreting the alt a1 family protein PevD1 to regulate the ORE1–ACS6 (1‐aminocyclopropane‐1‐carboxylate synthase) cascade and enhance ET production (Zhang *et al*., 2021). In addition, cytotoxicity also plays a critical role in inducing cell necrosis, as VdNLPs are constitutively expressed during later stages of infection (Santhanam *et al*., 2013), accelerating disease symptoms and the production of microsclerotia to begin a new disease cycle. *V. dahliae* effectors contribute to microsclerotia formation in senescing mesophyll tissue by warding off other fungal niche competitors (Snelders *et al*., 2020, 2021).

## IV. CONCLUSIONS

(1) Verticillium wilt caused by *V. dahliae* is a notorious vascular wilt disease that leads to chlorosis, stunting and wilting. Two notable hypotheses exist regarding the mechanisms underlying Verticillium wilt symptom development: vessel occlusion and direct toxicity through secreted toxins (Fradin & Thomma, 2006; Báidez *et al*., 2007). Vascular occlusion occurs as a result of the biomass of the pathogen present in the plant xylem, in addition to defensive tylose or colloid production by the plant (Báidez *et al*., 2007). The toxin hypothesis suggests that the pathogen delivers complex compounds into its host, including large polymers of polysaccharides, protein‐lipopolysaccharides, glycoproteins, enzymes, etc., that induce foliar wilting *via* cytotoxicity (Fradin & Thomma, 2006; de Sain & Rep, 2015).

(2) Similar to other plant pathogens, the secretome of *V. dahliae* has evolved to alter host structure and physiology, facilitating colonization. Specifically, components of the secretome of *V. dahliae* degrade plant cell walls, manipulate host immunity, exert cytotoxicity, neutralize host oxidative stresses, compete with its host for nutrients, and alter host endophytic associations and the rhizosphere microbiome, finally contributing to Verticillium wilt.

(3) Except for some *V. dahliae* secretome members with direct cytotoxicity, *V. dahliae* mainly employs its secretome to support proliferation in the plant xylem. These activities contribute to xylem wall collapse and the deposition of polysaccharides, and induction of host plant responses that promote vascular occlusion, finally compromising the host by causing total vascular occlusion. Thus, the *V. dahliae* secretome manipulates plant defences to contribute to vascular occlusion simultaneously with its toxicity effects to cause wilt symptoms.

(4) There are several key phases in the *V. dahliae*–plant interaction in which the weapons available in the secretome play critical roles in the induction of Verticillium wilt symptoms. In the initial infection zone in the roots, these effector activities specifically counter plant defences, neutralize oxidative stress, manipulate host immunity, and interfere with hormone homeostasis. Subsequently, in the vascular battlefield, the secretome aids mycelial proliferation, the release of incompletely degraded polysaccharides from plant cell walls and the induction of biomacromolecules from an active host defence response, all of which contribute to vascular occlusion. Final victory in shoot occurs as the toxic functions of the secretome manipulate immunity and hormone homeostasis to aggravate wilt symptoms further.

(5) Description of the functions of the secretory proteins in detail will allow their exact roles in vascular occlusion and toxicity to be clarified. Unravelling the bridge that links the *V. dahliae* secretome to the historical vascular occlusion and toxin hypotheses will shed new light into novel means to control this and related plant vascular pathogens.

## References

[brv12863-bib-0001] Báidez, A. G. , Gómez, P. , Río, J. A. D. & Ortuño, A. (2007). Dysfunctionality of the xylem in *Olea europaea* L. plants associated with the infection process by *Verticillium dahliae* Kleb. Role of phenolic compounds in plant defense mechanism. Journal of Agricultural and Food Chemistry 55, 3373–3377. 17394331 10.1021/jf063166d

[brv12863-bib-0002] Bailey, B. A. , James, C. J. & James, D. A. (1997). The 24‐kDa protein from *Fusarium oxysporum* f.sp. erythroxyli: occurrence in related fungi and the effect of growth medium on its production. Canadian Journal of Microbiology 43, 45–55. 9057295 10.1139/m97-007

[brv12863-bib-0003] Benhamou, N. (1995). Ultrastructural and cytochemical aspects of the response of eggplant parenchyma cells in direct contact with *Verticillium*‐infected xylem vessels. Physiological and Molecular Plant Pathology 46, 321–338.

[brv12863-bib-0004] Broxton, C. N. & Culotta, V. C. (2016). SOD enzymes and microbial pathogens: surviving the oxidative storm of infection. PLoS Pathogens 12, e1005295. 26742105 10.1371/journal.ppat.1005295 PMC4712152

[brv12863-bib-0005] Chavarro‐Carrero, E. A. , Vermeulen, J. P. , Torres, D. E. , Usami, T. , Schouten, H. J. , Bai, Y. , Seidl, M. F. & Thomma, B. P. H. J. (2021). Comparative genomics reveals the in planta‐secreted *Verticillium dahliae* Av2 effector protein recognized in tomato plants that carry the V2 resistance locus. Environmental Microbiology 23, 1941–1958. 33078534 10.1111/1462-2920.15288 PMC8246953

[brv12863-bib-0006] Chen, J. Y. , Klosterman, S. J. , Hu, X. P. , Dai, X. F. & Subbarao, K. V. (2021). Key insights and research prospects at the dawn of the population genomics era for *Verticillium dahliae* . Annual Review of Phytopathology 59, 31–51. 10.1146/annurev-phyto-020620-121925 33891830

[brv12863-bib-0007] Chen, J. Y. , Liu, C. , Gui, Y. J. , Si, K. W. , Zhang, D. D. , Wang, J. , Short, D. P. G. , Huang, J. Q. , Li, N. Y. , Liang, Y. , Zhang, W. Q. , Yang, L. , Ma, X. F. , Li, T. G. , Zhou, L. , et al. (2018). Comparative genomics reveals cotton‐specific virulence factors in flexible genomic regions in *Verticillium dahliae* and evidence of horizontal gene transfer from *Fusarium* . New Phytologist 217, 756–770. 29084346 10.1111/nph.14861 PMC5765495

[brv12863-bib-0008] Chen, J. Y. , Xiao, H. L. , Gui, Y. J. , Zhang, D. D. , Li, L. , Bao, Y. M. & Dai, X. F. (2016). Characterization of the *Verticillium dahliae* exoproteome involves in pathogenicity from cotton‐containing medium. Frontiers in Microbiology 7, 1709. 27840627 10.3389/fmicb.2016.01709 PMC5083787

[brv12863-bib-0009] Chu, X. Q. , Chen, W. , Chen, X. B. , Lu, W. J. , Han, L. , Wang, X. L. , Hao, L. L. & Guo, X. Q. (2015). The cotton WRKY gene *GhWRKY41* positively regulates salt and drought stress tolerance in transgenic *Nicotiana benthamiana* . PLoS One 10, e0143022. 26562293 10.1371/journal.pone.0143022 PMC4643055

[brv12863-bib-0010] Clérivet, A. , Déon, V. , Alami, I. , Lopez, F. , Geiger, J. P. & Nicole, M. (2000). Tyloses and gels associated with cellulose accumulation in vessels are responses of plane tree seedlings (*Platanus × acerifolia*) to the vascular fungus *Ceratocystis fimbriata* f. sp *platani* . Trees‐Structure and Function 15, 25–31.

[brv12863-bib-0011] de Jonge, R. , van Esse, H. P. , Maruthachalam, K. , Bolton, M. D. , Santhanam, P. , Saber, M. K. , Zhang, Z. , Usami, T. , Lievens, B. , Subbarao, K. V. & Thomma, B. P. H. J. (2012). Tomato immune receptor Ve1 recognizes effector of multiple fungal pathogens uncovered by genome and RNA sequencing. Proceedings of the National Academy of Sciences of the United States of America 109, 5110–5115. 22416119 10.1073/pnas.1119623109 PMC3323992

[brv12863-bib-0012] de Sain, M. & Rep, M. (2015). The role of pathogen‐secreted proteins in fungal vascular wilt diseases. International Journal of Molecular Sciences 16, 23970–23993. 26473835 10.3390/ijms161023970 PMC4632733

[brv12863-bib-0013] Dhar, N. , Chen, J. Y. , Subbarao, K. V. & Klosterman, S. J. (2020). Hormone signaling and its interplay with development and defense responses in *Verticillium*‐plant interactions. Frontiers in Plant Science 11, 584997. 33250913 10.3389/fpls.2020.584997 PMC7672037

[brv12863-bib-0014] Ding, P. T. & Redkar, A. (2018). Pathogens suppress host transcription factors for rampant proliferation. Trends in Plant Science 23, 950–953. 30241734 10.1016/j.tplants.2018.08.010

[brv12863-bib-0015] El‐Bebany, A. F. , Rampitsch, C. & Daayf, F. (2010). Proteomic analysis of the phytopathogenic soilborne fungus *Verticillium dahliae* reveals differential protein expression in isolates that differ in aggressiveness. Proteomics 10, 289–303. 20017145 10.1002/pmic.200900426

[brv12863-bib-0016] Fradin, E. F. & Thomma, B. P. H. J. (2006). Physiology and molecular aspects of Verticillium wilt diseases caused by *V. dahliae* and *V. albo‐atrum* . Molecular Plant Pathology 7, 71–86. 20507429 10.1111/j.1364-3703.2006.00323.x

[brv12863-bib-0017] Gao, F. , Zhang, B. S. , Zhao, J. H. , Huang, J. F. , Jia, P. S. , Wang, S. , Zhang, J. , Zhou, J. M. & Guo, H. S. (2019). Deacetylation of chitin oligomers increases virulence in soil‐borne fungal pathogens. Nature Plants 5, 1167–1176. 31636399 10.1038/s41477-019-0527-4

[brv12863-bib-0018] Glass, N. L. , Schmoll, M. , Cate, J. & Coradetti, S. (2013). Plant cell wall deconstruction by ascomycete fungi. Annual Review of Microbiology 67, 477–498. 10.1146/annurev-micro-092611-150044 23808333

[brv12863-bib-0019] Gui, Y. J. , Chen, J. Y. , Zhang, D. D. , Li, N. Y. , Li, T. G. , Zhang, W. Q. , Wang, X. Y. , Short, D. P. G. , Li, L. , Guo, W. , Kong, Z. Q. , Bao, Y. M. , Subbarao, K. V. & Dai, X. F. (2017). *Verticillium dahliae* manipulates plant immunity by glycoside hydrolase 12 proteins in conjunction with carbohydrate‐binding module 1. Environmental Microbiology 19, 1914–1932. 28205292 10.1111/1462-2920.13695

[brv12863-bib-0020] Gui, Y. J. , Zhang, W. Q. , Zhang, D. D. , Zhou, L. , Short, D. P. G. , Wang, J. , Ma, X. F. , Li, T. G. , Kong, Z. Q. , Wang, B. L. , Wang, D. L. , Li, N.‐Y. , Subbarao, K. V. , Chen, J. Y. & Dai, X. F. (2018). A *Verticillium dahliae* extracellular cutinase modulates plant immune responses. Molecular Plant‐Microbe Interactions 31, 260–273. 29068240 10.1094/MPMI-06-17-0136-R

[brv12863-bib-0021] Han, L. B. , Li, Y. B. , Wang, F. X. , Wang, W. Y. & Xia, G. X. (2019). The cotton apoplastic protein CRR1 stabilizes chitinase 28 to facilitate defense against the fungal pathogen *Verticillium dahliae* . Plant Cell 31, 520–536. 30651348 10.1105/tpc.18.00390 PMC6447012

[brv12863-bib-0022] Harholt, J. (2010). Biosynthesis of pectin. Plant Physiology 153, 384–395. 20427466 10.1104/pp.110.156588 PMC2879803

[brv12863-bib-0023] He, Q. , Mclellan, H. , Boevink, P. C. & Birch, P. R. J. (2020). All roads lead to susceptibility: the many modes of action of fungal and oomycete intracellular effectors. Plant Communications 1, 100050. 33367246 10.1016/j.xplc.2020.100050 PMC7748000

[brv12863-bib-0024] Heinz, R. , Lee, S. W. , Saparno, A. , Nazar, R. N. & Robb, J. (1998). Cyclical systemic colonization in *Verticillium*‐infected tomato. Physiological and Molecular Plant Pathology 52, 385–396.

[brv12863-bib-0025] Hogenhout, S. A. , van der Hoorn, R. A. , Terauchi, R. & Kamoun, S. (2009). Emerging concepts in effector biology of plant‐associated organisms. Molecular Plant‐Microbe Interactions 22, 115–222. 19132864 10.1094/MPMI-22-2-0115

[brv12863-bib-0026] Hu, Q. , Min, L. , Yang, X. Y. , Jin, S. X. , Zhang, L. , Li, Y. Y. , Ma, Y. Z. , Qi, X. W. , Li, D. Q. , Liu, H. B. , Lindsey, K. , Zhu, L. F. & Zhang, X. L. (2018). Laccase GhLac1 modulates broad‐spectrum biotic stress tolerance via manipulating phenylpropanoid pathway and jasmonic acid synthesis. Plant Physiology 176, 1808–1823. 29229698 10.1104/pp.17.01628 PMC5813555

[brv12863-bib-0027] Hu, X. P. , Puri, K. D. , Gurung, S. , Klosterman, S. J. & Subbarao, K. V. (2019). Proteome and metabolome analyses reveal differential responses in tomato–*Verticillium dahliae*‐interactions. Journal of Proteomics 207, 103449. 31323424 10.1016/j.jprot.2019.103449

[brv12863-bib-0028] Inderbitzin, P. & Subbarao, K. V. (2014). *Verticillium* systematics and evolution: how confusion impedes Verticillium wilt management and how to resolve it. Phytopathology 104, 564–574. 24548214 10.1094/PHYTO-11-13-0315-IA

[brv12863-bib-0029] Jing, J. , Fan, L. W. & Wu, W. H. (2005). Evidences for involvement of endogenous cAMP in *Arabidopsis* defense responses to *Verticillium* toxins. Cell Research 15, 585–592. 16117848 10.1038/sj.cr.7290328

[brv12863-bib-0030] Khademian, M. & Imlay, J. A. (2021). How microbes evolved to tolerate oxygen. Trends in Microbiology 29, 428–440. 33109411 10.1016/j.tim.2020.10.001 PMC8043972

[brv12863-bib-0031] Klimes, A. & Dobinson, K. F. (2006). A hydrophobin gene, *VDH1*, is involved in microsclerotial development and spore viability in the plant pathogen *Verticillium dahliae* . Fungal Genetics and Biology 43, 283–294. 16488633 10.1016/j.fgb.2005.12.006

[brv12863-bib-0032] Klimes, A. , Dobinson, K. F. , Thomma, B. P. H. J. & Klosterman, S. J. (2015). Genomics spurs rapid advances in our understanding of the biology of vascular wilt pathogens in the genus *Verticillium* . Annual Review of Phytopathology 53, 181–198. 10.1146/annurev-phyto-080614-120224 26047557

[brv12863-bib-0033] Klosterman, S. J. , Subbarao, K. V. , Kang, S. , Veronese, P. , Gold, S. E. , Thomma, B. P. H. J. , Chen, Z. , Henrissat, B. , Lee, Y. H. , Park, J. , et al. (2011). Comparative genomics yields insights into niche adaptation of plant vascular wilt pathogens. PLoS Pathogens 7, e1002137. 21829347 10.1371/journal.ppat.1002137 PMC3145793

[brv12863-bib-0034] Kombrink, A. , Rovenich, H. , Shi‐Kunne, X. Q. , Rojas‐Padilla, E. , van den Berg, G. C. M. , Domazakis, E. , de Jonge, R. , Valkenburg, D. J. , Sánchez‐Vallet, A. , Seidl, M. F. & Thomma, B. P. H. J. (2017). *Verticillium dahliae* LysM effectors differentially contribute to virulence on plant hosts. Molecular Plant Pathology 18, 596–608. 27911046 10.1111/mpp.12520 PMC6638240

[brv12863-bib-0035] Kubicek, C. P. , Starr, T. L. & Glass, N. L. (2014). Plant cell wall‐degrading enzymes and their secretion in plant‐pathogenic fungi. Annual Review of Phytopathology 52, 427–451. 10.1146/annurev-phyto-102313-045831 25001456

[brv12863-bib-0036] Lenarčič, T. , Albert, I. , Böhm, H. , Hodnik, V. , Pirc, K. , Zavec, A. B. , Podobnik, M. , Pahovnik, D. , Žagar, E. , Pruitt, R. , Greimel, P. , Yamaji‐Hasegawa, A. , Kobayashi, T. , Zienkiewicz, A. , Gömann, J. , et al. (2017). Eudicot plant‐specific sphingolipids determine host selectivity of microbial NLP cytolysins. Science 358, 1431–1143. 29242345 10.1126/science.aan6874

[brv12863-bib-0037] Leśniewska, J. , Öhman, D. , Krzesłowska, M. , Kushwah, S. , Barciszewska‐Pacak, M. , Kleczkowski, L. A. , Sundberg, B. , Moritz, T. & Mellerowicz, E. J. (2017). Defense responses in aspen with altered pectin methylesterase activity reveal the hormonal inducers of Tyloses. Plant Physiology 173, 1409–1419. 27923986 10.1104/pp.16.01443 PMC5291032

[brv12863-bib-0038] Li, N. X. & Kang, S. C. (2018). Do volatile compounds produced by *Fusarium oxysporum* and *Verticillium dahliae* affect stress tolerance in plants? Mycology 9, 166–175. 30181923 10.1080/21501203.2018.1448009 PMC6115880

[brv12863-bib-0039] Liang, Y. B. , Cui, S. C. , Tang, X. L. , Zhang, Y. , Qiu, D. W. , Zeng, H. M. , Guo, L. H. , Yuan, J. J. & Yang, X. F. (2018). An asparagine‐rich protein Nbnrp1 modulate *Verticillium dahliae* protein PevD1‐induced cell death and disease resistance in *Nicotiana benthamiana* . Frontiers in Plant Science 9, 303. 29563924 10.3389/fpls.2018.00303 PMC5846053

[brv12863-bib-0040] Liu, S. Y. , Chen, J. Y. , Wang, J. L. , Li, L. , Xiao, H. L. , Adam, S. M. & Dai, X. F. (2013). Molecular characterization and functional analysis of a specific secreted protein from highly virulent defoliating *Verticillium dahliae* . Gene 529, 307–316. 23891822 10.1016/j.gene.2013.06.089

[brv12863-bib-0041] Liu, T. L. , Song, T. Q. , Zhang, X. , Yuan, H. B. , Su, L. M. , Li, W. L. , Xu, J. , Liu, S. H. , Chen, L. L. , Chen, T. Z. , Zhang, M. X. , Gu, L. C. , Zhang, B. L. & Dou, D. L. (2014). Unconventionally secreted effectors of two filamentous pathogens target plant salicylate biosynthesis. Nature Communications 5, 4686. 10.1038/ncomms5686 PMC4348438 25156390

[brv12863-bib-0042] Liu, Y. K. , Tao, Q. , Li, J. X. , Guo, X. Y. , Luo, J. P. , Jupa, R. , Liang, Y. C. & Li, T. Q. (2020). Ethylene‐mediated apoplastic barriers development involved in cadmium accumulation in root of hyperaccumulator *Sedum alfredii* . Journal of Hazardous Materials 403, 123729. 33264898 10.1016/j.jhazmat.2020.123729

[brv12863-bib-0043] Ma, A. F. , Zhang, D. P. , Wang, G. X. , Wang, K. , Li, Z. , Gao, Y. H. , Li, H. C. , Bian, C. , Cheng, J. K. , Han, Y. N. , Yang, S. H. , Gong, Z. Z. & Qi, J. S. (2021). *Verticillium dahliae* effector VDAL protects MYB6 from degradation by interacting with PUB25 and PUB26 E3 ligases to enhance Verticillium wilt resistance. The Plant Cell 33, 3675–3699. 34469582 10.1093/plcell/koab221 PMC8643689

[brv12863-bib-0044] Martens‐Uzunova, E. S. & Schaap, P. J. (2009). Assessment of the pectin degrading enzyme network of *Aspergillus niger* by functional genomics. Fungal Genetics and Biology 46, S170–S179. 19618506 10.1016/j.fgb.2008.07.021

[brv12863-bib-0045] Maruthachalam, K. , Klosterman, S. J. , Kang, S. , Hayes, R. J. & Subbarao, K. V. (2011). Identification of pathogenicity‐related genes in the vascular wilt fungus *Verticillium dahliae* by *Agrobacterium tumefaciens*‐mediated T‐DNA insertional mutagenesis. Molecular Biotechnology 49, 209–221. 21424547 10.1007/s12033-011-9392-8 PMC3183274

[brv12863-bib-0046] Meyer, R. & Dubery, I. A. (1993). High‐affinity binding of a protein‐lipopolysaccharide phytotoxin from *Verticillium dahliae* to cotton membranes. FEBS Letters 335, 203–206. 8253197 10.1016/0014-5793(93)80730-i

[brv12863-bib-0047] Mohnen, D. (2008). Pectin structure and biosynthesis. Current Opinion in Plant Biology 11, 266–277. 18486536 10.1016/j.pbi.2008.03.006

[brv12863-bib-0048] Nachmias, A. , Buchner, V. & Burstein, Y. (1985). Biological and immunochemical characterization of a low molecular weight phytotoxin isolated from a protein–lipopolysaccharide complex produced by a potato isolate of *Verticillium dahliae* Kleb. Physiological Plant Pathology 26, 43–55.

[brv12863-bib-0049] Ottmann, C. , Luberacki, B. , Küfner, I. , Koch, W. , Brunner, F. , Weyand, M. , Mattinen, L. , Pirhonen, M. , Anderluh, G. , Seitz, H. U. , Nürnberger, T. & Oecking, C. (2009). A common toxin fold mediates microbial attack and plant defense. Proceedings of the National Academy of Sciences of the United States of America 106, 10359–10364. 19520828 10.1073/pnas.0902362106 PMC2695407

[brv12863-bib-0050] Palmer, C. S. , Saleeba, J. A. & Lyon, B. R. (2005). Phytotoxicity on cotton ex‐plants of an 18.5 kDa protein from culture filtrates of *Verticillium dahliae* . Physiological and Molecular Plant Pathology 67, 308–318.

[brv12863-bib-0051] Pegg, G. F. & Brady, B. L. (2002). Verticillium Wilts. CABI Publ, New York.

[brv12863-bib-0052] Qi, J. , Wang, J. , Gong, Z. & Zhou, J. M. (2017). Apoplastic ROS signaling in plant immunity. Current Opinion in Plant Biology 38, 92–100. 28511115 10.1016/j.pbi.2017.04.022

[brv12863-bib-0053] Qin, J. , Wang, K. L. , Sun, L. F. , Xing, H. Y. , Wang, S. , Li, L. , Chen, S. , Guo, H. S. & Zhang, J. (2018). The plant‐specific transcription factors CBP60g and SARD1 are targeted by a *Verticillium* secretory protein VdSCP41 to modulate immunity. Elife 7, e34902. 29757140 10.7554/eLife.34902 PMC5993538

[brv12863-bib-0054] Reusche, M. , Thole, K. , Janz, D. , Truskina, J. , Rindfleisch, S. , Drübert, C. , Polle, A. , Lipka, V. & Teichmann, T. (2012). *Verticillium* infection triggers VASCULAR‐RELATED NAC DOMAIN7‐dependent dependent de novo xylem formation and enhances drought tolerance in *Arabidopsis* . The Plant Cell 24, 3823–3837. 23023171 10.1105/tpc.112.103374 PMC3480305

[brv12863-bib-0055] Rioux, D. , Nicole, M. , Simard, M. & Ouellette, G. B. (1998). Immunocytochemical evidence that secretion of pectin occurs during gel (Gum) and tylosis formation in trees. Phytopathology 88, 494–505. 18944900 10.1094/PHYTO.1998.88.6.494

[brv12863-bib-0056] Robb, J. , Brisson, J. D. , Busch, L. & Lu, B. C. (1979). Ultrastructure of wilt syndrome caused by *Verticillium dahliae*. VII. Correlated light and transmission electron microscope identification of vessel coatings and tyloses. Canadian Journal of Botany 57, 822–834.

[brv12863-bib-0057] Robb, J. , Powell, D. A. & Street, P. (1989). Vascular coating: a barrier to colonization by the pathogen in Verticillium wilt of tomato. Canadian Journal of Botany 67, 600–607.

[brv12863-bib-0058] Rovenich, H. , Boshoven, J. C. & Thomma, B. P. H. J. (2014). Filamentous pathogen effector functions: of pathogens, hosts and microbiomes. Current Opinion in Plant Biology 20, 96–103. 24879450 10.1016/j.pbi.2014.05.001

[brv12863-bib-0059] Santhanam, P. , van Esse, H. P. , Albert, I. , Faino, L. , Nurnberger, T. & Thomma, B. P. H. J. (2013). Evidence for functional diversification within a fungal NEP1‐like protein family. Molecular Plant‐Microbe Interactions 26, 278–286. 23051172 10.1094/MPMI-09-12-0222-R

[brv12863-bib-0060] Schreiber, L. (2010). Transport barriers made of cutin, suberin and associated waxes. Trends in Plant Science 15, 546–553. 20655799 10.1016/j.tplants.2010.06.004

[brv12863-bib-0061] Shigenaga, A. M. & Argueso, C. T. (2016). No hormone to rule them all: interactions of plant hormones during the responses of plants to pathogens. Seminars in Cell & Developmental Biology 56, 174–189. 27312082 10.1016/j.semcdb.2016.06.005

[brv12863-bib-0062] Snelders, N. C. , Kettles, G. J. , Rudd, J. J. & Thomma, B. P. H. J. (2018). Plant pathogen effector proteins as manipulators of host microbiomes? Molecular Plant Pathology 19, 257–259. 29368817 10.1111/mpp.12628 PMC5817402

[brv12863-bib-0063] Snelders, N. C. , Petti, G. C. , van den Berg, G. C. M. , Seidl, M. F. & Thomma, B. P. H. J. (2021). An ancient antimicrobial protein co‐opted by a fungal plant pathogen for in planta mycobiome manipulation. Proceedings of the National Academy of Sciences of the United States of America 118, e2110968118. 34853168 10.1073/pnas.2110968118 PMC8670511

[brv12863-bib-0064] Snelders, N. C. , Rovenich, H. , Petti, G. C. , Rocafort, M. , van den Berg, G. C. M. , Vorholt, J. A. , Mesters, J. R. , Seidl, M. F. , Nijland, R. & Thomma, B. P. H. J. (2020). Microbiome manipulation by a soil‐borne fungal plant pathogen using effector proteins. Nature Plants 6, 1365–1374. 33139860 10.1038/s41477-020-00799-5

[brv12863-bib-0065] Stoddart, J. L. & Carr, A. J. H. (1966). Properties of wilt‐toxins produced by *Verticillium albo‐atrum* R & B. Annals of Applied Biology 58, 81–92.

[brv12863-bib-0066] Sun, Q. , Rost, T. L. , Reid, M. S. & Matthews, M. A. (2007). Ethylene and not embolism is required for wound‐induced tylose development in stems of grapevines. Plant Physiology 145, 1629–1636. 17921344 10.1104/pp.107.100537 PMC2151685

[brv12863-bib-0067] Tian, L. , Huang, C. M. , Zhang, D. D. , Li, R. , Chen, J. Y. , Sun, W. X. , Qiu, N. W. & Dai, X. F. (2021a). Extracellular superoxide dismutase VdSOD5 is required for virulence in *Verticillium dahliae* . Journal of Integrative Agriculture 20, 1858–1870.

[brv12863-bib-0068] Tian, L. , Li, J. J. , Huang, C. M. , Zhang, D. D. , Xu, Y. , Yang, X. Y. , Song, J. , Wang, D. , Qiu, N. W. , Short, D. P. G. , Inderbitzin, P. , Subbarao, K. V. , Chen, J. Y. & Dai, X. F. (2021b). Cu/Zn superoxide dismutase (VdSOD1) mediates reactive oxygen species detoxification and modulates virulence in *Verticillium dahliae* . Molecular Plant Pathology 22, 1092–1108. 34245085 10.1111/mpp.13099 PMC8359004

[brv12863-bib-0069] Tian, L. , Sun, W. X. , Li, J. J. , Chen, J. Y. , Dai, X. F. , Qiu, N. W. & Zhang, D. D. (2020). Unconventionally secreted manganese superoxide dismutase VdSOD3 is required for the virulence of *Verticillium dahliae* . Agronomy 11, 13.

[brv12863-bib-0070] Trapero, C. , Alcántara, E. , Jiménez, J. , Amaro‐Ventura, M. C. , Romero, J. , Koopmann, B. , Karlovsky, P. , Tiedemann, A. V. , Pérez‐Rodríguez, M. & López‐Escudero, F. J. (2018). Starch hydrolysis and vessel occlusion related to wilt symptoms in olive stems of susceptible sultivars infected by *Verticillium dahliae* . Frontiers in Plant Science 9, 72. 29445388 10.3389/fpls.2018.00072 PMC5797883

[brv12863-bib-0071] Tzima, A. K. , Paplomatas, E. J. , Rauyaree, P. , Ospinagiraldo, M. D. & Kang, S. (2021). VdSNF1, the sucrose nonfermenting protein kinase gene of *Verticillium dahliae*, is required for virulence and expression of genes involved in cell‐wall degradation. Molecular Plant‐Microbe Interactions 24, 129–142. 10.1094/MPMI-09-09-0217 20839958

[brv12863-bib-0072] Wang, B. N. , Yang, X. F. , Zeng, H. M. , Liu, H. , Zhou, T. T. , Tan, B. B. , Yuan, J. J. , Guo, L. H. & Qiu, D. W. (2012). The purification and characterization of a novel hypersensitive‐like response‐inducing elicitor from *Verticillium dahliae* that induces resistance responses in tobacco. Applied Microbiology and Biotechnology 93, 191–201. 21691787 10.1007/s00253-011-3405-1

[brv12863-bib-0073] Wang, C. H. , Wang, H. , Li, P. X. , Li, H. Y. , Xu, C. M. , Cohen, H. , Aharoni, A. & Wu, S. (2020b). Developmental programs interact with abscisic acid to coordinate root suberization in *Arabidopsis* . Plant Journal 104, 241–251. 10.1111/tpj.14920 32645747

[brv12863-bib-0074] Wang, D. , Chen, J. Y. , Song, J. , Li, J. J. , Klosterman, S. J. , Li, R. , Kong, Z. Q. , Subbarao, K. V. , Dai, X. F. & Zhang, D. D. (2021a). Novel cytotoxic function of xylanase VdXyn4 in the plant vascular wilt pathogen *Verticillium dahliae* . Plant Physiology 187, 409–429. 34618145 10.1093/plphys/kiab274 PMC8418393

[brv12863-bib-0075] Wang, D. , Tian, L. , Zhang, D. D. , Song, J. , Song, S. S. , Yin, C. M. , Zhou, L. , Liu, Y. , Wang, B. L. , Kong, Z. Q. , Klosterman, S. , Li, J. J. , Wang, J. , Li, T. G. , Adamu, S. , et al. (2020a). Functional analyses of small secreted cysteine‐rich proteins identified candidate effectors in *Verticillium dahliae* . Molecular Plant Pathology 21, 667–685. 32314529 10.1111/mpp.12921 PMC7170778

[brv12863-bib-0076] Wang, D. , Zhang, D. D. , Usami, T. , Liu, L. , Yang, L. , Huang, J. Q. , Song, J. , Li, R. , Kong, Z. Q. , Li, J. J. , Wang, J. , Klosterman, S. J. , Subbarao, K. V. , Dai, X. F. & Chen, J. Y. (2021b). Functional genomics and comparative lineage‐specific region analyses reveal novel insights into race divergence in *Verticillium dahliae* . Microbiology Spectrum 9, e0111821. 34937170 10.1128/Spectrum.01118-21 PMC8694104

[brv12863-bib-0077] Wang, J. Y. , Cai, Y. , Gou, J. Y. , Mao, Y. B. , Xu, Y. H. , Jiang, W. H. & Chen, X. Y. (2004). VdNEP, an elicitor from *Verticillium dahliae*, induces cotton plant wilting. Applied Microbiology and Biotechnology 70, 4989–4995. 10.1128/AEM.70.8.4989-4995.2004 PMC492334 15294839

[brv12863-bib-0078] Xie, C. J. , Li, Q. L. & Yang, X. Y. (2017). Characterization of VdASP F2 secretory factor from *Verticillium dahliae* by a fast and easy gene knockout system. Molecular Plant‐Microbe Interactions 30, 444–454. 28291379 10.1094/MPMI-01-17-0007-R

[brv12863-bib-0079] Xiong, D. G. , Wang, Y. L. & Tian, C. M. (2015). Transcriptomic profiles of the smoke tree wilt fungus *Verticillium dahliae* under nutrient starvation stresses. Molecular Genetics and Genomics 290, 1963–1977. 25939502 10.1007/s00438-015-1052-4

[brv12863-bib-0080] Yadeta, K. A. & Thomma, B. P. H. J. (2013). The xylem as battleground for plant hosts and vascular wilt pathogens. Frontiers in Plant Science 4, 97. 23630534 10.3389/fpls.2013.00097 PMC3632776

[brv12863-bib-0081] Yang, Y. K. , Zhang, Y. , Li, B. B. , Yang, X. F. , Dong, Y. J. & Qiu, D. W. (2018). A *Verticillium dahliae* pectate lyase induces plant immune responses and contributes to virulence. Frontiers in Plant Science 9, 1271. 30271415 10.3389/fpls.2018.01271 PMC6146025

[brv12863-bib-0082] Yin, C. M. , Li, J. J. , Wang, D. , Zhang, D. D. , Song, J. , Kong, Z. Q. , Wang, B. L. , Hu, X. P. , Klosterman, S. J. , Subbarao, K. V. , Chen, J. Y. & Dai, X. F. (2022). A secreted ribonuclease effector from *Verticillium dahliae* localizes in the plant nucleus to modulate host immunity. Molecular Plant Pathology. 10.1111/mpp.13213. PMC9276946 35363930

[brv12863-bib-0083] Yin, Z. , Wang, N. , Pi, L. , Li, L. , Duan, W. , Wang, X. & Dou, D. L. (2021). *Nicotiana benthamiana* LRR‐RLP NbEIX2 mediates the perception of an EIX‐like protein from *Verticillium dahliae* . Journal of Integrative Plant Biology 63, 949–960. 33205907 10.1111/jipb.13031

[brv12863-bib-0084] Zhang, D. D. , Wang, J. , Wang, D. , Kong, Z. Q. , Zhou, L. , Zhang, G. Y. , Gui, Y. J. , Li, J. J. , Huang, J. Q. , Wang, B. L. , Liu, C. , Yin, C. M. , Li, R. X. , Li, T. G. , Wang, J. L. , et al. (2019a). Population genomics demystifies the defoliation phenotype in the plant pathogen *Verticillium dahliae* . New Phytologist 22, 1012–1029. 10.1111/nph.15672 PMC6594092 30609067

[brv12863-bib-0085] Zhang, D. D. , Wang, X. Y. , Chen, J. Y. , Kong, Z. Q. & Dai, X. F. (2016). Identification and characterization of a pathogenicity‐related gene *VdCYP1* from *Verticillium dahliae* . Scientific Reports 6, 27979. 27329129 10.1038/srep27979 PMC4916405

[brv12863-bib-0086] Zhang, J. , Zhang, Y. , Yang, J. , Kang, L. , Elorm, A. M. , Zhou, H. Y. & Zhao, J. (2019b). The α‐1,6‐mannosyltransferase VdOCH1 plays a major role in microsclerotium formation and virulence in the soil‐borne pathogen *Verticillium dahliae* . Fungal Biology 123, 539–546. 31196523 10.1016/j.funbio.2019.05.007

[brv12863-bib-0087] Zhang, L. S. , Ni, H. , Du, X. , Wang, S. , Ma, X. W. , Nürnberger, T. , Guo, H. S. & Hua, C. L. (2017b). The *Verticillium*‐specific protein VdSCP7 localizes to the plant nucleus and modulates immunity to fungal infections. New Phytologist 215, 368–381. 28407259 10.1111/nph.14537

[brv12863-bib-0088] Zhang, W. Q. , Gui, Y. J. , Short, D. P. G. , Li, T. G. , Zhang, D. D. , Zhou, L. , Liu, C. , Bao, Y. M. , Subbarao, K. V. , Chen, J. Y. & Dai, X. F. (2018). *Verticillium dahliae* transcription factor VdFTF1 regulates the expression of multiple secreted virulence factors and is required for full virulence in cotton. Molecular Plant Pathology 19, 841–857. 28520093 10.1111/mpp.12569 PMC6638078

[brv12863-bib-0089] Zhang, Y. , Gao, Y. H. , Liang, Y. B. , Dong, Y. J. , Yang, X. F. , Yuan, J. J. & Qiu, D. W. (2017a). The *Verticillium dahliae* SnodProt1‐Like protein VdCP1 contributes to virulence and triggers the plant immune system. Frontiers in Plant Science 8, 1880. 29163605 10.3389/fpls.2017.01880 PMC5671667

[brv12863-bib-0090] Zhang, Y. , Gao, Y. H. , Wang, H. L. , Kan, C. C. , Li, Z. , Yang, X. F. , Yin, W. L. , Xia, X. L. , Nam, H. G. , Li, Z. H. & Guo, H. W. (2021). *Verticillium dahliae* secretory effector PevD1 induces leaf senescence by promoting ORE1‐mediated ethylene biosynthesis. Molecular Plant 14, 1901–1917. 34303024 10.1016/j.molp.2021.07.014

[brv12863-bib-0091] Zhao, J. , Chen, Q. H. , Zhou, S. , Sun, Y. H. & Li, Y. Z. (2020). H2Bub1 regulates RbohD‐dependent hydrogen peroxide signal pathway in the defense responses to *Verticillium dahliae* toxins. Plant Physiology 182, 640–657. 31666300 10.1104/pp.19.00913 PMC6945848

[brv12863-bib-0092] Zhao, P. , Zhao, Y. L. , Jin, Y. , Zhang, T. & Guo, H. S. (2014). Colonization process of *Arabidopsis thaliana* roots by a green fluorescent protein‐tagged isolate of *Verticillium dahliae* . Protein & Cell 5, 94–98. 24481631 10.1007/s13238-013-0009-9 PMC3956967

[brv12863-bib-0093] Zhao, Y. L. , Zhou, T. T. & Guo, H. S. (2016). Hyphopodium‐specific VdNoxB/VdPls1‐dependent ROS‐Ca2+ signaling is required for plant infection by *Verticillium dahliae* . PLoS Pathogens 12, e1005793. 27463643 10.1371/journal.ppat.1005793 PMC4962994

[brv12863-bib-0094] Zhou, B. J. , Jia, P. S. , Gao, F. & Guo, H. S. (2012). Molecular characterization and functional analysis of a necrosis‐ and ethylene‐inducing, protein‐encoding gene family from *Verticillium dahliae* . Molecular Plant‐Microbe Interactions 25, 964–975. 22414440 10.1094/MPMI-12-11-0319

[brv12863-bib-0095] Zhou, T. T. , Zhao, Y. L. & Guo, H. S. (2017). Secretory proteins are delivered to the septin‐organized penetration interface during root infection by *Verticillium dahliae* . PLoS Pathogens 13, e1006275. 28282450 10.1371/journal.ppat.1006275 PMC5362242

